# Prognostic nutritional index as a predictive marker for acute kidney injury in adult critical illness population: a systematic review and diagnostic test accuracy meta-analysis

**DOI:** 10.1186/s40560-024-00729-z

**Published:** 2024-04-26

**Authors:** Jia-Jin Chen, Tao-Han Lee, Pei-Chun Lai, Chih-Hsiang Chang, Che-Hsiung Wu, Yen-Ta Huang

**Affiliations:** 1 Kidney Research Center, Department of Nephrology, Chang Gung Memorial Hospital, Linkou Branch, Chang Gung university, Taoyuan, 33305 Taiwan; 2 Department of Nephrology, Chansn Hospital, Taoyuan City, 33305 Taiwan; 3 Education Center, National Cheng Kung University Hospital, College of Medicine, National Cheng Kung University, Tainan, Taiwan; 4 Division of Nephrology, Taipei Tzu Chi Hospital, Buddhist Tzu Chi Medical Foundation, New Taipei City, 231 Taiwan; 5 School of Medicine, Tzu Chi University, Hualien, 970 Taiwan; 6 Department of Surgery, National Cheng Kung University Hospital, College of Medicine, National Cheng Kung University, No. 138, Shengli Road, Tainan, 701 Taiwan

**Keywords:** Acute kidney injury, Prognostic nutritional index, Meta-analysis, Risk

## Abstract

**Background:**

The prognostic nutritional index (PNI), integrating nutrition and inflammation markers, has been increasingly recognized as a prognostic predictor in diverse patient cohorts. Recently, its effectiveness as a predictive marker for acute kidney injury (AKI) in various clinical settings has gained attention. This study aims to assess the predictive accuracy of the PNI for AKI in critically ill populations through systematic review and meta-analysis.

**Methods:**

A systematic review was conducted using the databases MEDLINE, EMBASE, PubMed, and China National Knowledge Infrastructure up to August 2023. The included trials reported the PNI assessment in adult population with critical illness and its predictive capacity for AKI. Data on study characteristics, subgroup covariates, and diagnostic performance of PNI, including sensitivity, specificity, and event rates, were extracted. A diagnostic test accuracy meta-analysis was performed. Subgroup analyses and meta-regression were utilized to investigate the sources of heterogeneity. The GRADE framework evaluated the confidence in the meta-analysis’s evidence.

**Results:**

The analysis encompassed 16 studies with 17 separate cohorts, totaling 21,239 patients. The pooled sensitivity and specificity of PNI for AKI prediction were 0.67 (95% CI 0.58–0.74) and 0.74 (95% CI 0.67–0.80), respectively. The pooled positive likelihood ratio was 2.49 (95% CI 1.99–3.11; low certainty), and the negative likelihood ratio was 0.46 (95% CI 0.37–0.56; low certainty). The pooled diagnostic odds ratio was 5.54 (95% CI 3.80–8.07), with an area under curve of summary receiver operating characteristics of 0.76. Subgroup analysis showed that PNI’s sensitivity was higher in medical populations than in surgical populations (0.72 vs. 0.55; *p* < 0.05) and in studies excluding patients with chronic kidney disease (CKD) than in those including them (0.75 vs. 0.56; *p* < 0.01). Overall, diagnostic performance was superior in the non-chronic kidney disease group.

**Conclusion:**

Our study demonstrated that PNI has practical accuracy for predicting the development of AKI in critically ill populations, with superior diagnostic performance observed in medical and non-CKD populations. However, the diagnostic efficacy of the PNI has significant heterogeneity with different cutoff value, indicating the need for further research.

**Supplementary Information:**

The online version contains supplementary material available at 10.1186/s40560-024-00729-z.

## Background

Acute kidney injury (AKI) is a frequent complication in patients with critically illness, with varying incidences across patient groups. For instance, post-major surgery patients show AKI rates between 3 and 35% [1]; patients with myocardial infarction, with or without percutaneous coronary intervention (PCI), show AKI rates ranging from 7 to 27% [2, 3]; and cardiac surgery participants show AKI rates between 19 and 32% [4, 5]. Multiple factors, such as sepsis, pre-existing diabetes mellitus (DM) or cardiovascular disease, chronic kidney disease (CKD), advanced age, anemia, and hypoalbuminemia, contribute to AKI susceptibility [6–8].

The prognostic nutritional index (PNI), initially developed by Buzby [9] and later modified by Onodera and Kosaki [10], is a readily accessible marker evaluating nutritional and inflammatory status. This index is calculated as 10 × serum albumin (g/dL) + 0.005 × total lymphocyte count (/mm^3^). It has been linked to post-operative or peri-treatment morbidity and mortality across various patient groups, including those with various malignancy [11–13], heart failure [14], and DM [15]. Subsequent research has identified PNI as a prognostic factor for outcomes or AKI risk assessment in populations with critically illness, including acute coronary syndrome [16–18], major abdominal surgery [19], ICU admissions [20], and COVID-19 infection [21]. Kurtul et al. demonstrated that a PNI of less than 38 has 82% sensitivity and 70% specificity for contrast-associated AKI prediction in patients with ST-segment elevation myocardial infarction who underwent percutaneous coronary intervention [16]. Shimoyama showed that a PNI with a cutoff point of 26.08 has 58% sensitivity and 67% specificity for sepsis AKI prediction among enrolled ICU patients with sepsis [22].

Despite the abovementioned studies, a systematic examination of the diagnostic accuracy for PNI as an AKI risk stratification tool is lacking. In the current study, a systematic review and meta-analysis was conducted to (1) analyze the diagnostic accuracy of PNI as an AKI risk stratification tool and (2) determine whether the diagnostic accuracy of PNI could be affected by predefined covariates such as different populations and underlying CKD.

## Materials and methods

### Literature search strategy

This meta-analysis was performed in accordance with Preferred Reporting Items for Systematic Reviews and Meta-Analyses (PRISMA) for Diagnostic Test Accuracy (DTA) Studies [23] (Additional file 1: Table S1). The protocol was registered with PROSPERO (CRD42023456607, registration date: 01 September 2023). Two independent reviewers (J.J. Chen and T.H. Lee) conducted a comprehensive systematic review and searched for articles published until August 07, 2023, in PubMed, MEDLINE, EMBASE, and China National Knowledge Infrastructure (CNKI). The search strategy relied on the use of the following keywords: [acute kidney injury], [acute renal failure], and [prognostic nutritional index]. Detailed search strategies are provided in Additional file 1: Table S2. Review articles were not included in the present analysis; however, the references of these studies were screened for relevant studies. The language or article types had no limitations.

### Study eligibility criteria

The titles and abstracts of the studies extracted from the search were independently examined by two reviewers (Chen and Lee), and articles were excluded during initial screening if the titles or abstracts indicated that they were clearly irrelevant to the objective of the current study. The full texts of the relevant articles were reviewed to examine whether the studies were eligible for inclusion.

A study was included if it met the criteria of adult humans as its population and reported the cutoff point of PNI and its diagnostic/predictive ability for AKI development. The other inclusion criteria were that the study reported at least one of the following outcomes of interest:

1. Occurrence rate of AKI with various definitions (either guideline-based AKI criteria, such as Acute Kidney Injury Network [AKIN], Kidney Disease: Improving Global Outcomes [KDIGO], European Society of Urogenital Radiology [ESUR], or predefined AKI criteria by individual studies);
2. Sensitivity and specificity based on a defined cutoff value of PNI for AKI prediction or with a receiver operating characteristic (ROC) curve of PNI for AKI prediction.

A third reviewer (Y.T. Huang) was consulted to reach an agreement through consensus in the case of any disagreement regarding eligibility. Studies were excluded if they were duplicate cohorts or had insufficient information about the outcomes.

### Data extraction and outcome measurement

Two investigators (Chen and Lee) independently extracted relevant information from each study. The extracted data elements included the first author, year of publication, study location, study design, patient source (coronary artery disease for percutaneous coronary intervention or cardiac surgery, liver transplant, or sepsis), AKI judgment timing and definition, the measured day of PNI, and whether the study excluded CKD population or not. As for diagnostic test performance, the extracted data included the cutoff value of PNI based on the Youden index or predefined criteria, sensitivity, specificity, number of true positive, number of false positive, and the event number of AKI (Tables 1 and 2). Disagreements about data extraction between the two authors (Chen and Lee) were resolved through discussion with a third author (Huang).

**Table 1:** The characteristics of the included studies

Study	Year of data collection	Study design	Country	Location	Enrolled participants	Mean age	%, Female	Excluded CKD^#^	AKI definition	AKI judgement timing	PNI cut-off value	PNI measure time	Mean serum albumin (g/L)
Aykut, 2022	2019 to 2020	Retrospective	Turkey	Single-center	On-pump CABG	61	17.8%	Excluding	KDIGO	1 wk post-OP	< 48	pre-OP	4.2
Dolapoglu, 2019	2015 to 2018	Retrospective	Turkey	Single-center	On-pump CABG	64	25.0%	Excluding	AKIN	Within 48 h post-OP	46.5	Pre-OP	3.7
Efe, 2021	NR	Retrospective	Turkey	NR	Chronic CAD	69	36.7%	Excluding	ESUR	72–96 h post-PCI	≤ 38	pre-PCI	3.1
Gucu, 2021	2016 to 2020	Retrospective	Turkey	Single-center	On-pump CABG	64	40.6%	Excluding	KDIGO	48 h post-OP	< 42.9	Pre-OP	3.8
Han, 2021	2010 to 2018	Retrospective	Korea	Single-center	PCI	65	28.4%	Including	KDIGO	Within 48 h post-PCI	< 47.8	Pre-PCI	4.2
Hatem, 2023	2019	Retrospective	Turkey	Single-center	Non-STEMI with PCI	62	27.1%	Excluding	≧ 25% or 0.3 mg/dL increase in baseline serum creatinine levels	Within 48–72 h Post-PCI	< 48.5	Pre-PCI	NR
Hu (test), 2021	NR	Retrospective	MIMIC III	Multi-center	CCU	67	59.2%	Including	KDIGO	Within 7 days post admission	< 48.8	1st day of admission	3.3
Hu (validation), 2021	2014 to 2015	Retrospective	China	Single-center	CCU	66	67.2%	Including	KDIGO	Within 7 days post admission	< 45.7	1st day of admission	3.7
Jing, 2021	2019	Retrospective	China	Single-center	Operation with CPB but not CABG	52	54.1%	Excluding	AKIN	Within 48 h post-OP	NR	Pre-OP	4.5
Kurtul, 2021	2017 to 2020	Retrospective	Turkey	Two-center	STEMI with PCI	58	24.0%	Excluding	ESUR	Within 72 h post-PCI	< 38	Pre-PCI	
Lee, 2019	2013 to 2016	Retrospective	Korea	Single-center	Cardiac surgery	59	43.0%	Including	Creatinine elevation of twofold the immediate preoperative value or the new requirement for renal replacement therapy	1 wk post-OP	≤ 46.13	Pre-OP	3.9
Li, 2022	2009 to 2019	Retrospective	China	Multi-center	PCI	67	34.0%	Excluding	ESUR	within 72-h	< 41.4	Pre-PCI	3.9
Min, 2020	2011 to 2018	Retrospective	Korea	Single-center	Living donor liver transplant	52	28.8%	Excluding	AKIN	1 wk post-OP	< 25	pre-OP	3.7
Shimoyama, 2021	2017 to 2019	Prospective	Japan	Single-center	Sepsis	74	38.6%	Including	KDIGO-3 (dialysis-requiring AKI)	2,3,5 day after ICU admission	< 19.51	1st day of ICU	2.3
Shimoyama, 2022	2017 to 2019	Prospective	Japan	Single-center	ICU paitents	75	35.6%	Including	KDIGO	2,3,5 day after ICU admissio	< 26.08	1st day of ICU	2.3
Xie, 2022	2015 to 2021	Retrospective	China	Single-center	Sepsis	58	36.2%	Excluding	KDIGO	As KDIGO criteria	< 32.75	1st day of admission	3.1
Yuksel, 2023	2016 to 2020	Retrospective	Turkey	Single-center	ACS with PCI	63	26.5%	Excluding	ESUR	Not reported	< 48.6	Pre-PCI	4

ACS: acute coronary syndrome; AKIN: Acute Kidney Injury Network; CABG: coronary artery bypass graft surgery; CAD: coronary artery disease; CCU: coronary care unit,; CKD: chronic kidney disease; CPB: cardiopulmonary bypass; ESUR: European Society of Urogenital Radiology; ICU: intensive care unit; KDIGO: Kidney Disease: Improving Global Outcomes; NR: non-reported; OP: operation; PCI: percutaneous coronary intervention;

^**#**^For studies that did not mention excluding chronic kidney disease (CKD) populations, or failed to report on this aspect, we categorized them as including CKD populations

**Table 2:** Diagnostic test performance of prognostic nutritional index for acute kidney injury in enrolled studies

Study	Total participants	Number of AKI	AUC	Sensitivity	Specificity	True positive	True negative	False positive	False negative
Aykut, 2022	455	141	NR	0.24	0.84	34	264	50	107
Dolapoglu, 2019	336	88	0.792 (0.728–0.856)	0.66	0.92	58	228	20	30
Efe, 2021	360	91	NR	0.52	0.80	47	216	53	44
Gucu, 2021	255	82	0.711 (0.649–0.774)	0.67	0.77	55	133	40	27
Han, 2021	3731	271	0.707	0.56	0.76	152	2638	822	119
Hatem, 2023	336	68	0.732	0.71	0.69	48	186	82	20
Hu (test), 2021	6444	4457	0.755 (0.734–0.775)	0.64	0.82	2830	1625	362	1627
Hu (validation), 2021	412	130	0.738 (0.685–0.791)	0.75	0.65	97	182	100	33
Jing, 2021	584	98	0.553 (0.489–0.617)	0.65	0.47	64	230	256	34
Kurtul, 2021	836	79	0.836 (0.788–0.805)	0.82	0.70	65	530	227	14
Lee, 2019	374	31	0.57	0.39	0.74	12	255	88	19
Li, 2022	4386	3599	0.629 (0.603–0.655)	0.80	0.40	630	1440	2159	157
Min, 2020	423	54	0.749 (0.705–0.790)	0.70	0.74	38	273	96	16
Shimoyama, 2021	83	6	0.72	0.67	0.94	4	72	5	2
Shimoyama, 2022	61	38	0.6	0.58	0.67	22	15	8	16
Xie, 2022	1238	507	0.760 (0.731–0.789)	0.87	0.64	441	466	265	66
Yuksel, 2023	925	232	0.87 (0.84–0.89)	0.80	0.81	188	554	139	44

AKI: acute kidney injury; NR: not reported

### Data synthesis and analysis

#### Summary of the effect

The AKI event numbers; total sample size; and true positive (TP), false positive (FP), true negative (TN), and false negative (FN) for PNI as an AKI prediction tool based on Youden index or predefined cutoff value were extracted. We also extracted these information by calculating from the reported sensitivity or specificity, or measured from ROC. The summary measures (pooled sensitivity, specificity, positive likelihood ratio [+ LR], negative likelihood ratio [− LR]) was calculated using a univariate model through the *metapro & metabin* function. The diagnostic odds ratio (DOR) was calculated using a bivariate model through the *metabin* function in R “meta” package. A random-effects model with maximum likelihood estimation was used to estimate the between-study variance. This model was selected due to the anticipated heterogeneity among the studies included in this analysis. The heterogeneity is attributed to variations in the study populations (surgical or medical), the procedures received, the baseline underlying diseases, and the different cutoff values for PNI. A summary receiver operating characteristic (SROC) curve with a bivariate model, which was established by the *reitsma* function in R “mada” package, was used to assess the predictive performance of PNI regarding AKI development.

#### Analysis of heterogeneity between studies

The threshold effect was examined using Spearman’s correlation coefficient test [24, 25]. A *p* value ≥ 0.6 indicated considerable threshold effect. If no significant threshold effect was found, subgroup analysis or meta-regression analysis was further performed to explore the sources of heterogeneity. Between-study variance (tau-squared) was evaluated through maximum likelihood estimation, and the result of heterogeneity examination was presented as the *I*^2^ index and *p* value of Chi-squared test. An *I*^2^ > 50% indicated substantial heterogeneity. Several potential covariates were identified, including patient population (population: medical vs. surgical patients, procedure: underwent PCI or not, including CKD or not, hypoalbuminemia or not [serum albumin level less than 3.5 g/L], and used AKI diagnosis criteria by KDIGO criteria or others). Subgroup analysis was performed to examine whether a difference existed in the diagnostic performance in different subgroups. Subgroup analysis was performed by employing the *subgroup* order within the *metaprop* and *metabin* functions to calculate the pooled sensitivity, specificity, positive likelihood ratio (LR), negative LR, and diagnostic odds ratio (DOR) across various subgroups. The overall PNI diagnostic performance between subgroups was examined by the relative DOR (RDOR). It was calculated using the *metareg* function in the “meta” package. Diagnostic odds ratio is a measure derived from comparing the odds of positive test outcomes among participants with the target disease to the odds of positive outcomes in those without the target disease [26, 27]. RDOR can be utilized to compare various diagnostic tests or evaluate diagnostic accuracy across different studies [28, 29]. In the subgroup analyses, a coefficient can estimate the change in the log-transformed DOR for studies featuring a specific covariate versus those without. By applying an antilogarithm transformation to this coefficient, yielding the RDOR, we can interpret this RDOR as the mean DOR for studies with a particular covariate relative to those without a particular covariate. An RDOR greater than 1 suggests enhanced diagnostic accuracy in studies that include the specific covariate [30].

Publication bias was assessed using a funnel plot and Deeks test through the *metabias* function [31]. All analyses were performed using R version 4.2.2 (2022-10-31) [32]. A two-sided *p* value of < 0.05 indicated statistically significant.

### Risk of bias and certainty of evidence assessment

The risk of bias for each included study was assessed using the Quality Assessment of Diagnostic Accuracy Studies 2 (QUADAS-2) tool. The judgment of risk of bias in the QUADAS-2 tool is based on four main domains: patient selection, index test, reference standard, and flow and timing [33]. If all signaling questions for a domain receive “yes” responses, the domain is deemed to have a low risk of bias. Conversely, an answer of “no” to any question in a domain indicates a high risk of bias. Two independent reviewers (Chen and Lee) rated high, low, or unclear risk for four domains, and disagreements between the reviewers were resolved by discussion with another author (Huang). The judgment of “applicability” followed the same principle as in the bias section, but no signaling questions were asked. The certainty of evidence for the diagnostic performance of PNI for AKI prediction was evaluated on the basis of the guidelines of the GRADE Working Group methodology [34–36].

## Results

### Search result and study characteristics

A flowchart of the literature search is provided in Additional file 1: Fig. S1. The electronic database search identified 225 potentially eligible studies from PubMed, 83 from EMBASE (Excerpta Medica database), 27 from CNKI, and 23 from MEDLINE. The remaining 301 articles were screened after duplicate articles were removed. After the titles and abstracts were screened, the full texts of 28 studies were reviewed to assess their eligibility. After studies were excluded for various reasons (no outcome of interest [*n* = 11]; duplication cohort [*n* = 1], Additional file 1: Table S2), 16 studies (17 separate cohorts) comprising 21,239 patients were included for analysis [16, 17, 22, 37–49].

The detailed characteristics of the enrolled studies are presented in Table 1. The enrolled patients had a mean age ranging from 52 to 75 years, and the average percentage of female participants was 36.6%. Fourteen of the 16 enrolled studies were retrospective trials, and the remaining two were prospective studies. Nearly half of the enrolled studies (seven out of 16) defined AKI by KDIGO criteria, followed by ESUR post-contrast criteria (*n* = 4) and the AKIN criteria (*n* = 3). Five of the 16 studies enrolled patients who underwent cardiac surgery, and the other six studies enrolled patients who underwent PCI.

### Prognostic nutritional index for AKI prediction

**Fig. 1:**
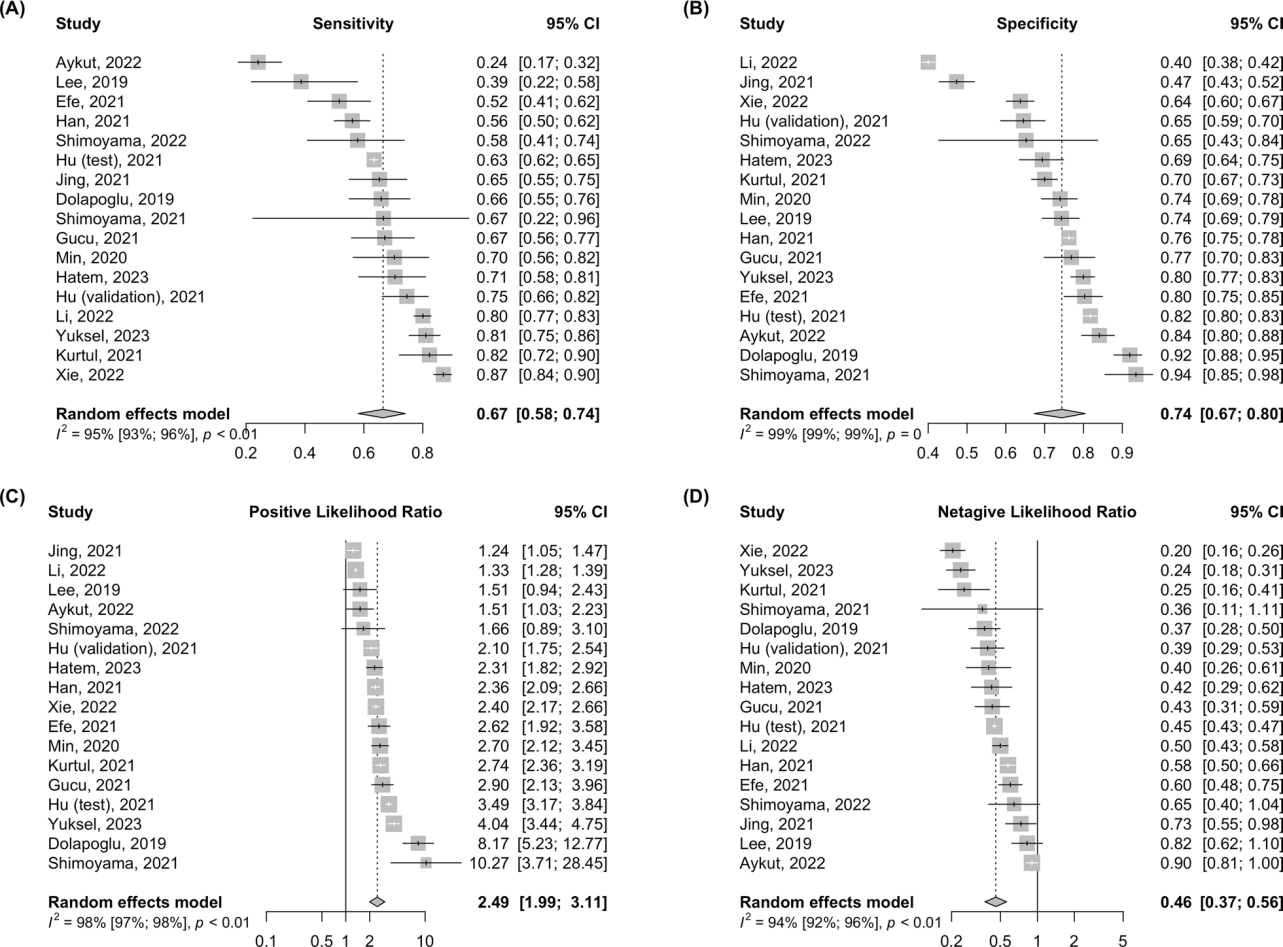
Forest plot of prognostic nutritional index diagnostic accuracy: **A** sensitivity, **B** specificity, **C** positive likelihood ratio, and **D** negative likelihood ratio for acute kidney injury

**Fig. 2:**
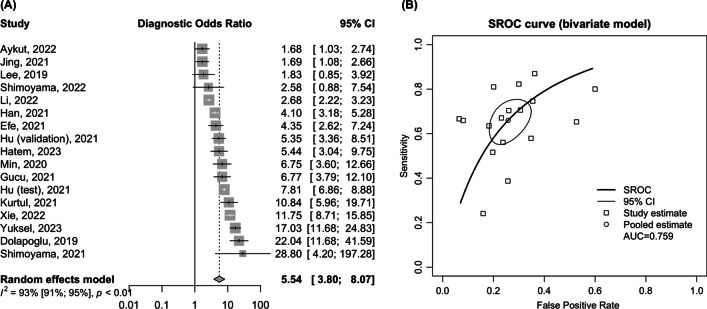
Pooled diagnostic odds ratio (**A**) and SROC curves (**B**) of prognostic nutritional index for prediction of AKI. AKI: acute kidney injury; SROC: summary receiver operating characteristic

**Fig. 3:**
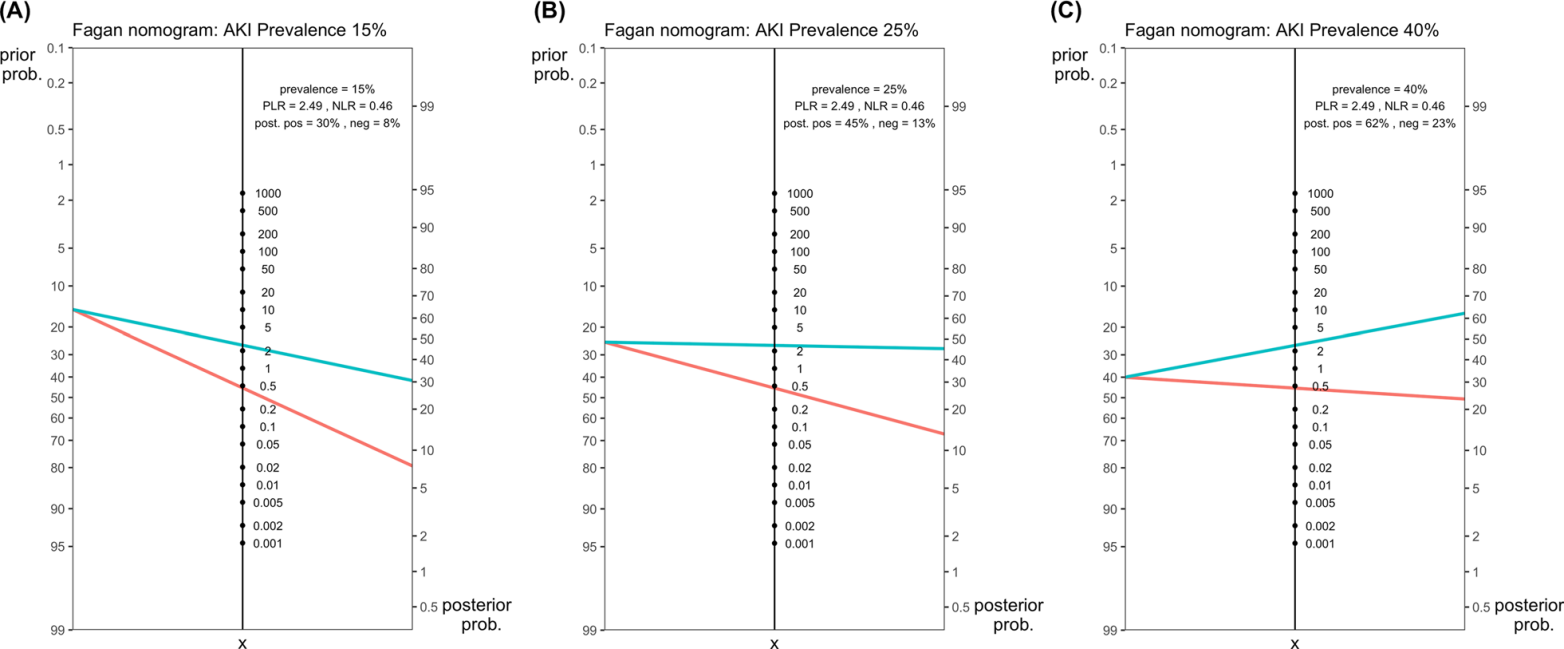
Fagan’s nomogram for prognostic nutritional index as acute kidney injury prediction marker with pre-test probabilities of 15% (**A**), 25% (**B**), and 40% (**C**)

The diagnostic value, cutoff points, and key results of the enrolled studies are summarized in Tables 1 and 2. The pooled sensitivity and specificity were 0.67 (95% CI 0.58–0.74) and 0.74 (95% CI 0.67–0.80), respectively, with substantial heterogeneity (Fig. 1). The pooled positive LR and negative LR were 2.49 (95% CI 1.99–3.11) and 0.46 (95% CI 0.37–0.56), respectively (Fig. 1). The pooled DOR was 5.54 (95% CI 3.80–8.07), with substantial heterogeneity (Fig. 2A). The area under the curve (AUC) for SROC to summarize the diagnostic accuracy was 0.759 (Fig. 2B). In populations with varying AKI prevalence—low (15%), moderate (25%), and high (40%)—the post-test probabilities of AKI for those with positive PNI results were 30%, 45%, and 62%, respectively; conversely, for individuals with negative PNI results, these probabilities were 8%, 13%, and 23%, respectively (Fig. 3).

### Subgroup analysis

Spearman’s correlation coefficient test was conducted with a p value of 0.44, which indicated no significant threshold effect. Subgroup analysis was further performed in population (surgical vs. medical), received procedure or not (PCI vs. non-PCI), baseline CKD (including or excluding CKD population), serum albumin level (hypoalbuminemia vs. not), and used AKI criteria (KDIGO vs. other). The results showed that the PNI had higher sensitivity and negative LR in the medical population and population after excluding patients with CKD (Table 3). The overall diagnostic performance evaluated by RDOR was significantly lower in studies including patients with CKD (RDOR: 0.44, 95% CI 0.22–0.48, *p* = 0.02, Table 3). The diagnostic performance was not significantly different in populations with hypoalbuminemia or not and the used AKI criteria. In non-CKD individuals, the post-test AKI probabilities for negative PNI results were 6%, 11%, and 19% for pre-test AKI prevalence rates of 15%, 25%, and 40%, respectively (Additional file 1: Fig. S2).

**Table 3:** Heterogeneity analysis by subgroup analysis and meta-regression for prognostic nutritional index as an acute kidney injury prediction marker

Variables	Subgroups (number of studies)	Sensitivity (95% CI)	Specificity (95% CI)	Positive LR (95% CI)	Negative LR (95% CI)	Diagnostic odds ratio (95% CI)	RDOR (95% CI)^#^; *p* value^#^
Population	Surgical (6)	0.55* (0.40–0.70)	0.77 (0.74–0.86)	2.36 (1.44–3.87)	0.58* (0.43–0.79)	4.13 (1.92–8.92)	0.63 (0.28–1.42); 0.27
	Medical (10; 11 cohort)	0.72* (0.64–0.79)	0.73 (0.64–0.80)	2.54 (2.06–3.12)	0.40* (0.32–0.50)	6.46 (4.47–9.33)	
Procedure	PCI (6)	0.72 (0.61–0.80)	0.70 (0.59–0.80)	2.42 (1.84–3.19)	0.42 (0.31–0.56)	5.91 (3.52–9.91)	1.12 (0.49–2.32); 0.79
	Other (10; 11 cohort)	0.63 (0.52–0.73)	0.77 (0.68–0.84)	2.55 (1.84–3.55)	0.48 (0.37–0.63)	5.32 (3.12–8.96)	
CKD	Including CKD (7; 8 cohort)	0.56** (0.43–0.67)	0.75 (0.65–0.84)	2.12 (1.55–2.88)	0.61** (0.49–0.76)	3.50 (2.15–5.62)	0.44 (0.22–0.88); 0.02
	Excluding CKD (9)	0.75** (0.67–0.81)	0.74 (0.64–0.82)	2.81 (2.12–3.72)	0.36** (0.29–0.46)	7.92 (5.15–12.18)	
Serum albumin^##^	Alb < 3.5 g/L (5)	0.68 (0.52–0.80)	0.79 (0.67–0.87)	2.84 (2.26–3.57)	0.42 (0.28–0.36)	7.19 (4.60–11.23)	1.52 (0.58–3.97); 0.39
	Alb ≥ 3.5 g/L (10)	0.64 (0.52–0.74)	0.73 (0.63–0.81)	2.31 (1.67–3.21)	0.50 (0.39–0.64)	4.68 (2.74–8.00)	
AKI criteria	KDIGO (7; 8 cohort)	0.63 (0.49–0.76)	0.77 (0.69–0.83)	2.49 (2.05–3.03)	0.4 (0.34–0.64)	5.45 (3.45–8.62)	0.98 (0.44–2.22); 0.39
	Other (9)	0.69 (0.61–0.77)	0.72 (0.60–0.81)	2.46 (1.73–3.50)	0.45 (0.35–0.59)	5.58 (3.16–9.88)	

Diagnostic indices, including sensitivity, specificity, positive likelihood ratio, negative likelihood ratio, and diagnostic odds ratio, are determined through subgroup analysis. The overall diagnostic accuracy of the Prognostic Nutritional Index across subgroups, distinguished by the presence or absence of specific covariates, is evaluated using meta-regression and presented as the relative diagnostic odds ratio

^**#**^Relative diagnostic odds ratio (RDOR) with 95% CI and *p* value are obtained from meta-regression which selective covariates are examined as moderator to compare the overall diagnostic performance between subgroups

Subgroup difference for four diagnostic accuracy indices (sensitivity, specificity, positive LR and negative LR) were examined by subgroup analysis. *p value for subgroup difference < 0.05; **p value for subgroup difference < 0.01

^**##**^Two studies did not report baseline serum albumin level were not included in this subgroup analysis (Hatem 2023; Kurtul 2021). One study with two cohorts was classified into different subgroup (Hu, 2021)

AKI: acute kidney injury; Alb: albumin; KDIGO: Kidney Disease Improving Global Outcomes; LR: likelihood ratio; RDOR: relative diagnostic odds ratio

### Risk-of-bias of enrolled studies and certainty of evidence

The publication bias assessment via Deeks’ funnel plot showed no significant publication bias, with a p value of 0.9 (Additional file 1: Fig. S3).

Potential biases were identified using the QUADAS-2 tool. In Domain 1, which assessed patient selection, three studies [39, 40, 43] were marked as having unclear concerns due to the exclusion of specific populations, such as those with heart failure or anemia. One study [22] included patients already diagnosed with septic AKI, raising questions about selection bias. In Domain 2, which evaluated the index test (PNI), two studies [37, 39] used predefined cutoff points, whereas the remaining 14 studies relied on cutoffs defined by the Youden index, categorizing them as high risk in this domain. In Domain 3, which assessed the reference test, one study [22] was flagged for unclear risk. This present study defined the outcome as stage 3 AKI requiring dialysis. Finally, in Domain 4, which focused on patient flow and the timing of outcome assessment, classified five studies as high risk or unclear risk due to inadequate AKI follow-up timing or failure to report specific time periods (Additional file 1: Fig. S4).

The certainty of evidence for the predictive performance of PNI in this meta-analysis was assessed. Due to the lack of predefined cutoff values in most studies, the interpretation of the index test was considered to be biased. As a result, the risk-of-bias domain was ranked as serious concern. Regarding the inconsistency domain, the meta-analysis revealed high heterogeneity. Furthermore, two out of five predefined subgroup analyses demonstrated a significant difference in sensitivity, and none of the five predefined subgroups showed a significant difference in specificity. Consequently, the inconsistency domain was ranked as serious concern. Other three domains (indirectness, imprecision, and publication bias) were ranked as not serious concern. The overall certainty of evidence was low. Table 4 summarizes the results.

**Table 4:** GRADE certainty of evidence assessment

Outcome	Number of studies (no. of patients)	Study design	Factors that may decrease certainty of evidence					Effect per 100 patients tested			Test accuracy CoE
			Risk of bias	Indirectness	Inconsistency	Imprecision	Publication bias	Pre-test probability of 15%	Pre-test probability of 25%	Pre-test probability of 35%	
True positives (patients with acute kidney injury)	16 studies 7160 patients	Cross-sectional (cohort type accuracy study)	Serious^a^	Not serious	Serious^b^	Not serious	None	10 (9 to 11)	17 (14 to 19)	23 (20 to 26)	⨁⨁◯◯ Low
False negatives (patients incorrectly classified as not having acute kidney injury)								5 (4 to 6)	8 (6 to 11)	12 (9 to 15)	
True negatives (patients without acute kidney injury)	16 studies 14,079 patients	Cross-sectional (cohort type accuracy study)	Serious^a^	Not serious	Serious^b^	Not serious	None	63 (57 to 68)	55 (50 to 60)	48 (44 to 52)	⨁⨁◯◯ Low
False positives (patients incorrectly classified as having acute kidney injury)								22 (17 to 28)	20 (15 to 25)	17 (13 to 21)	

^a^Most of the enrolled studies did not use pre-specific cutoff value but depend on the Youden index

^b^The *I*^2^ values for sensitivity and specificity were notably high, at 95% and 98%, respectively. Among the five predefined subgroup analyses, only two—population and chronic kidney disease (CKD)—demonstrated potential differences in sensitivity. However, none of the five predefined subgroup analyses revealed significant differences in specificity

## Discussion

Our analysis revealed that the PNI has pooled sensitivity and specificity of 0.67 and 0.74, respectively, with an area under the SROC curve of 0.76 for predicting the onset of acute kidney injury (AKI). Additionally, we demonstrated that PNI could serve as an effective risk stratification tool for identifying individuals at low risk for AKI in populations with a relatively low occurrence rate of AKI (Fig. 3). The diagnostic accuracy of our assessment remained consistent across various AKI criteria and two mean serum albumin level groups. Sensitivity appears to be higher in medical patients compared to surgical patients (0.72 vs. 0.55), potentially reflecting a higher baseline postoperative inflammatory status in surgical patients relative to medical patients [50] (26,126,129). Moreover, we found that the diagnostic accuracy is improved in the non-CKD population. It has a relative diagnostic odds ratio (RDOR) of 0.44 for studies including CKD populations versus those excluding CKD populations. The overall diagnostic performance of PNI is comparable to several novel biomarkers, such as serum Neutrophil Gelatinase-Associated Lipocalin (NGAL) (sensitivity 0.76 and specificity 0.79) and Liver-Type Fatty Acid-Binding Protein (L-FABP) (sensitivity 0.69 and specificity 0.81) [51]. The performance of PNI was also similar to the renal angina index, which has a sensitivity of 0.82 and specificity of 0.77 for predicting the onset of AKI post-major surgery [52]. We concluded that, compared to these novel biomarkers, PNI is routinely available without additional cost in daily clinical practice, making it a practical and cost-effective option for AKI risk assessment.

Acute kidney injury, being a complex and multifactorial condition, encompasses exposure risk factors and susceptibility factors. Old age; hypovolemia; anemia; and chronic disease with chronic inflammation, such as CKD, chronic liver disease, and DM, are considered as susceptibility factors [7]. Moreover, low serum albumin level was identified as a risk factor for AKI development in patients with critical illness [53] or those who had cardiac surgery [54]. PNI, created by Buzby et al., merges serum albumin and lymphocytes to indicate nutritional and inflammatory states and stress response, initially aiding in operative risk assessment and preoperative nutrition in gastrointestinal surgery [55–58]. Several studies [16, 17, 22, 37–49] have explored the predictive efficacy of PNI in assessing the risk of AKI in diverse clinical populations with critical illness (e.g., cardiac surgery, coronary artery disease, liver transplant, and sepsis), with different AKI criteria and diverse PNI cutoff values. We are the first to conduct a comprehensive meta-analysis examining the diagnostic accuracy of the PNI for AKI prediction. Our analysis demonstrates that the diagnostic accuracy of PNI remains consistent across various disease populations, different AKI criteria, and different levels of baseline serum albumin.

This study has several limitations. First, the risk of bias in the included studies was not negligible, primarily because most did not employ pre-specified PNI cutoff values. Additionally, certain studies specifically excluded conditions commonly observed in patients with critical illness, such as anemia (serum Hb level < 10) [40] and congestive heart failure [39, 40, 42]. Second, the analysis did not account for factors other than malnutrition that may contribute to lymphopenia, such as malignancy or autoimmune disorders [59]. Third, while the subgroup analysis revealed significant heterogeneity between studies involving patients with CKD and those that did not, as well as between medical and surgical cohorts, this high degree of heterogeneity warrants caution in interpreting the findings. Moreover, the absence of large-scale, prospective studies and a standardized PNI cutoff point for predicting AKI and its use as a prognostic marker necessitate further investigation. Finally, the majority of the studies analyzed were retrospective cohorts that used the Youden index to determine cutoff values, leading to a lower level of confidence in the evidence due to heterogeneity.

## Conclusion

PNI could serve as a readily available marker for identifying patients with critical illness at low risk for AKI development. It demonstrates enhanced predictive performance, particularly in non-chronic kidney disease and medical populations. Additional trials with larger sample sizes and high-quality study designs are needed, employing predefined cutoff values for more precise assessments, to further elucidate the diagnostic utility of PNI in AKI risk classification within clinical settings.

### Supplementary Information

**Additional file 1: Table S1.** Checklist for Preferred Reporting Items for Systematic Reviews and Meta-Analyses (PRISMA) for Diagnostic Test Accuracy (DTA) studies. **Table S2.** Search strategy for each database. **Table S3.** Reasons for excluding full-text screening studies. **Figure S1.** PRISMA flowchart. **Figure S2.** Fagan’s nomogram for prognostic nutritional index as acute kidney injury prediction marker in non-CKD population with pre-test probabilities of 15% (A), 25% (B), and 40% (C). **Figure S3.** Deek’s funnel plot. **Figure S4.** Assessment (A) and summary (B) of risk of bias and applicability concern.

## Data Availability

The datasets used in this meta-analysis are available from the corresponding author on reasonable request.

## Abbreviations

ACS: Acute coronary syndrome
AKI: Acute kidney injury
AKIN: Acute Kidney Injury Network
CABG: Coronary artery bypass graft surgery
CAD: Coronary artery disease
CKD: Chronic kidney disease
DOR: Diagnostic odds ratio
KDIGO: Kidney Disease: Improving Global Outcomes
LR: Likelihood ratio
NR: Non-reported
PNI: Prognostic nutritional index
RDOR: Relative diagnostic odds ratio
SROC: Summary receiver operating characteristic
