# Supplementary material for: Prognostic nutritional index as a predictive marker for acute kidney injury in adult critical illness population: a systematic review and diagnostic test accuracy meta-analysis
Source: J Intensive Care. 2024 Apr 26;12:16. doi: 10.1186/s40560-024-00729-z (PMC11046764; doi:10.1186/s40560-024-00729-z)
Supplement: Supplementary file 1 — Additional file 1: Table S1. Checklist for Preferred Reporting Items for Systematic Reviews and Meta-Analyses (PRISMA) for Diagnostic Test Accuracy (DTA) studies. Table S2. Search strategy for each database. Table S3. Reasons for excluding full-text screening studies. Figure S1. PRISMA flowchart. Figure S2. Fagan’s nomogram for prognostic nutritional index as acute kidney injury prediction marker in non-CKD population with pre-test probabilities of 15% (A), 25% (B), and 40% (C). Figure S3. Deek’s funnel plot. Figure S4. Assessment (A) and summary (B) of risk of bias and applicability concern. [file 40560_2024_729_MOESM1_ESM.docx]

**Additional file**

**Table S1.** Checklist for Preferred Reporting Items for Systematic Reviews and Meta-Analyses (PRISMA) for Diagnostic Test Accuracy (DTA) Studies

**Table S2.** Search strategy for each database

**Table S3.** Reasons for excluding full-text screening studies

**Additional Figure Legend**

**Figure S1.** PRISMA flow chart

**Figure S2.** Fagan's nomogram for prognostic nutritional index as acute kidney injury prediction marker in non-CKD population with pre-test probability 15% (A), 25% (B) and 40% (C)

**Figure S3.** Deek’s funnel plot

**Figure S4.** Assessment (A) and Summary (B) of risk of bias and applicability concern

**Table S1.** Checklist for Preferred Reporting Items for Systematic Reviews and Meta-Analyses (PRISMA) for Diagnostic Test Accuracy (DTA) Studies

| **Section/topic** | **#** | **PRISMA-DTA Checklist Item** | **Reported on page #** |
| --- | --- | --- | --- |
| **TITLE / ABSTRACT** | | |  |
| Title | 1 | Identify the report as a systematic review (+/- meta-analysis) of diagnostic test accuracy (DTA) studies.3 | 1 |
| Abstract | 2 | Abstract: See PRISMA-DTA for abstracts. | 3 |
| **INTRODUCTION** | | |  |
| Rationale | 3 | Describe the rationale for the review in the context of what is already known. | 4 |
| Clinical role of index test | D1 | State the scientific and clinical background, including the intended use and clinical role of the index test, and if applicable, the rationale for minimally acceptable test accuracy (or minimum difference in accuracy for comparative design). | 4 |
| Objectives | 4 | Provide an explicit statement of question(s) being addressed in terms of participants, index test(s), and target condition(s). | 4 |
| **METHODS** | | |  |
| Protocol and registration | 5 | Indicate if a review protocol exists, if and where it can be accessed (e.g., Web address), and, if available, provide registration information including registration number. | 4,5 |
| Eligibility criteria | 6 | Specify study characteristics (participants, setting, index test(s), reference standard(s), target condition(s), and study design) and report characteristics (e.g., years considered, language, publication status) used as criteria for eligibility, giving rationale. | 5 |
| Information sources | 7 | Describe all information sources (e.g., databases with dates of coverage, contact with study authors to identify additional studies) in the search and date last searched. | 5 |
| Search | 8 | Present full search strategies for all electronic databases and other sources searched, including any limits used, such that they could be repeated. | 5 |
| Study selection | 9 | State the process for selecting studies (i.e., screening, eligibility, included in systematic review, and, if applicable, included in the meta-analysis). | 5 |
| Data collection process | 10 | Describe method of data extraction from reports (e.g., piloted forms, independently, in duplicate) and any processes for obtaining and confirming data from investigators. | 5 |
| Definitions for data extraction | 11 | Provide definitions used in data extraction and classifications of target condition(s), index test(s), reference standard(s) and other characteristics (e.g. study design, clinical setting). | 5 |
| Risk of bias and applicability | 12 | Describe methods used for assessing risk of bias in individual studies and concerns regarding the applicability to the review question. | 6 |
| Diagnostic accuracy measures | 13 | State the principal diagnostic accuracy measure(s) reported (e.g. sensitivity, specificity) and state the unit of assessment (e.g. per-patient, per-lesion). | 6 |
| Synthesis of results | 14 | Describe methods of handling data, combining results of studies and describing variability between studies. This could include, but is not limited to: a) handling of multiple definitions of target condition. b) handling of multiple thresholds of test positivity, c) handling multiple index test readers, d) handling of indeterminate test results, e) grouping and comparing tests, f) handling of different reference standards | 6 |
| Section/topic | # | PRISMA-DTA Checklist Item | Reported on page # |
| Meta-analysis | D2 | Report the statistical methods used for meta-analyses, if performed. | 5-6 |
| Additional analyses | 16 | Describe methods of additional analyses (e.g., sensitivity or subgroup analyses, meta-regression), if done, indicating which were pre-specified. | 6 |
| **RESULTS** | | |  |
| Study selection | 17 | Provide numbers of studies screened, assessed for eligibility, included in the review (and included in meta-analysis, if applicable) with reasons for exclusions at each stage, ideally with a flow diagram. | 7 |
| Study characteristics | 18 | For each included study provide citations and present key characteristics including: a) participant characteristics (presentation, prior testing), b) clinical setting, c) study design, d) target condition definition, e) index test, f) reference standard, g) sample size, h) funding sources | 7 |
| Risk of bias and applicability | 19 | Present evaluation of risk of bias and concerns regarding applicability for each study. | 8 |
| Results of individual studies | 20 | For each analysis in each study (e.g. unique combination of index test, reference standard, and positivity threshold) report 2x2 data (TP, FP, FN, TN) with estimates of diagnostic accuracy and confidence intervals, ideally with a forest or receiver operator characteristic (ROC) plot. | Table 1-2 |
| Synthesis of results | 21 | Describe test accuracy, including variability; if meta-analysis was done, include results and confidence intervals. | Figure 1 |
| Additional analysis | 23 | Give results of additional analyses, if done (e.g., sensitivity or subgroup analyses, meta-regression; analysis of index test: failure rates, proportion of inconclusive results, adverse events). | 7-8 |
| **DISCUSSION** | | |  |
| Summary of evidence | 24 | Summarize the main findings including the strength of evidence. | 8-9 |
| Limitations | 25 | Discuss limitations from included studies (e.g. risk of bias and concerns regarding applicability) and from the review process (e.g. incomplete retrieval of identified research). | 10 |
| Conclusions | 26 | Provide a general interpretation of the results in the context of other evidence. Discuss implications for future research and clinical practice (e.g. the intended use and clinical role of the index test). | 10 |
| **FUNDING** | | |  |
| Funding | 27 | For the systematic review, describe the sources of funding and other support and the role of the funders. | 11 |

**Table S2.** Search strategy for each database

| **Medline through Aug 08^st^, 2023**  #1 Prognostic Nutritional Index.mp.  #2 acute kidney injury.mp. or Acute Kidney Injury/  #3 acute renal failure.mp. or Acute Kidney Injury/  #4 2 OR 3  #5 1 AND 4  Result: 23 |
| --- |
| **EMbase through Aug 08^st^, 2023**  #1 'prognostic nutritional index'/exp OR 'prognostic nutritional index' OR (prognostic AND nutritional AND ('index'/exp OR index)  #2 acute AND kidney AND injury  #3 acute AND renal AND failure  #4 2 OR 3  #5 #1 AND #4  Result: 83 |
| **Pubmed through Aug 08^st^, 2023**  #1 Prognostic Nutritional Index  #2 acute kidney injury  #3 acute renal failure  #4 (acute kidney injury) OR (acute renal failure)  #5 (Prognostic Nutritional Index) AND ((acute kidney injury) OR (acute renal failure))  #6 (Prognostic Nutritional Index) AND ((acute kidney injury) OR (acute renal failure)) Filters: Humans  Result: 225 |
| **CNKI through Aug 08^st^, 2023**  #1 Prognostic Nutritional Index AND acute kidney injury  Result: 27 |

**Footnote:** In our study, 'acute kidney injury,' 'acute renal failure,' and 'prognostic nutritional index' were utilized as free-text keywords. Moreover, 'acute kidney injury' was indexed as a MeSH term in both PubMed and Medline databases.

**Table S3.** Reasons for excluding full-text screening studies

| **Author, year** | **Title** | **Reason for exclusion** | **Result** |
| --- | --- | --- | --- |
| 1.Akıllı, 2019 | Prognostic nutritional index and the risk of acute kidney injury in patients with acute coronary syndrome undergoing emergency percutaneous coronary intervention | No outcome of interest | Exclusion |
| 2.Acarbaş, 2021 | Which Objective Nutritional Index Is Better for the Prediction of Adverse Medical Events in Elderly Patients Undergoing Spinal Surgery? | No outcome of interest | Exclusion |
| 3.Bansal, 2021 | Preoperative Combined Adiposity–Nutritional Index Predicts Major aDverse Cardiac and Cerebral Events following Off-pump coRonary Artery Revascularization (PANDORA): A Retrospective Single-Center Study | No outcome of interest | Exclusion |
| 4.Cheng, 2017 | Prognostic Nutritional Index and the Risk of Mortality in Patients With Acute Heart Failure | No outcome of interest | Exclusion |
| 5.Dong, 2021 | Association between Prognostic Nutritional Index and Contrast-Associated Acute Kidney Injury in Patients Complicated with Chronic Kidney Disease and Coronary Artery Disease | No outcome of interest | Exclusion |
| 6.Kilic, 2022 | Prognostic nutritional index predicts perioperative adverse events in patients undergoing hemiarthroplasty after a hip fracture | No outcome of interest | Exclusion |
| 7.Li, 2020 | Malnutrition screening and acute kidney injury in hospitalised patients: a retrospective study over a 5-year period from China | No outcome of interest | Exclusion |
| 8.Lin, 2019 | Predictive value of objective nutritional scores for contrast-induced acute kidney injury in elderly patients undergoing percutaneous coronary intervention | No outcome of interest | Exclusion |
| 9.Sertdemir, 2021 | Prognostic nutritional index and the risk of acute kidney injury in patients with acute coronary syndrome | No outcome of interest | Exclusion |
| 10.Shimoyama, 2021 | Presepsin Values Predict Septic Acute Kidney Injury, Acute Respiratory Distress Syndrome, Disseminated Intravascular Coagulation, and Shock | Duplication cohort | Exclusion |
| 11.Sim, 2021 | Association of Preoperative Prognostic Nutritional Index and Postoperative Acute Kidney Injury in Patients with Colorectal Cancer Surgery | No outcome of interest | Exclusion |
| 12.Sim, 2021 | Association of Preoperative Prognostic Nutritional Index and Postoperative Acute Kidney Injury in Patients Who Underwent Hepatectomy for Hepatocellular Carcinoma | No outcome of interest | Exclusion |
| 陈希源 | 原位肝移植术后早期急性肾损伤的一种新预测模型及其围手术期的高危因素 | Not retrieved |  |
| 敬维维 | 术前预后营养指数对584例心脏手术后患者急性肾损伤的预测价值 | Not retrieved |  |

**Figure S1.** PRISMA flow chart

**
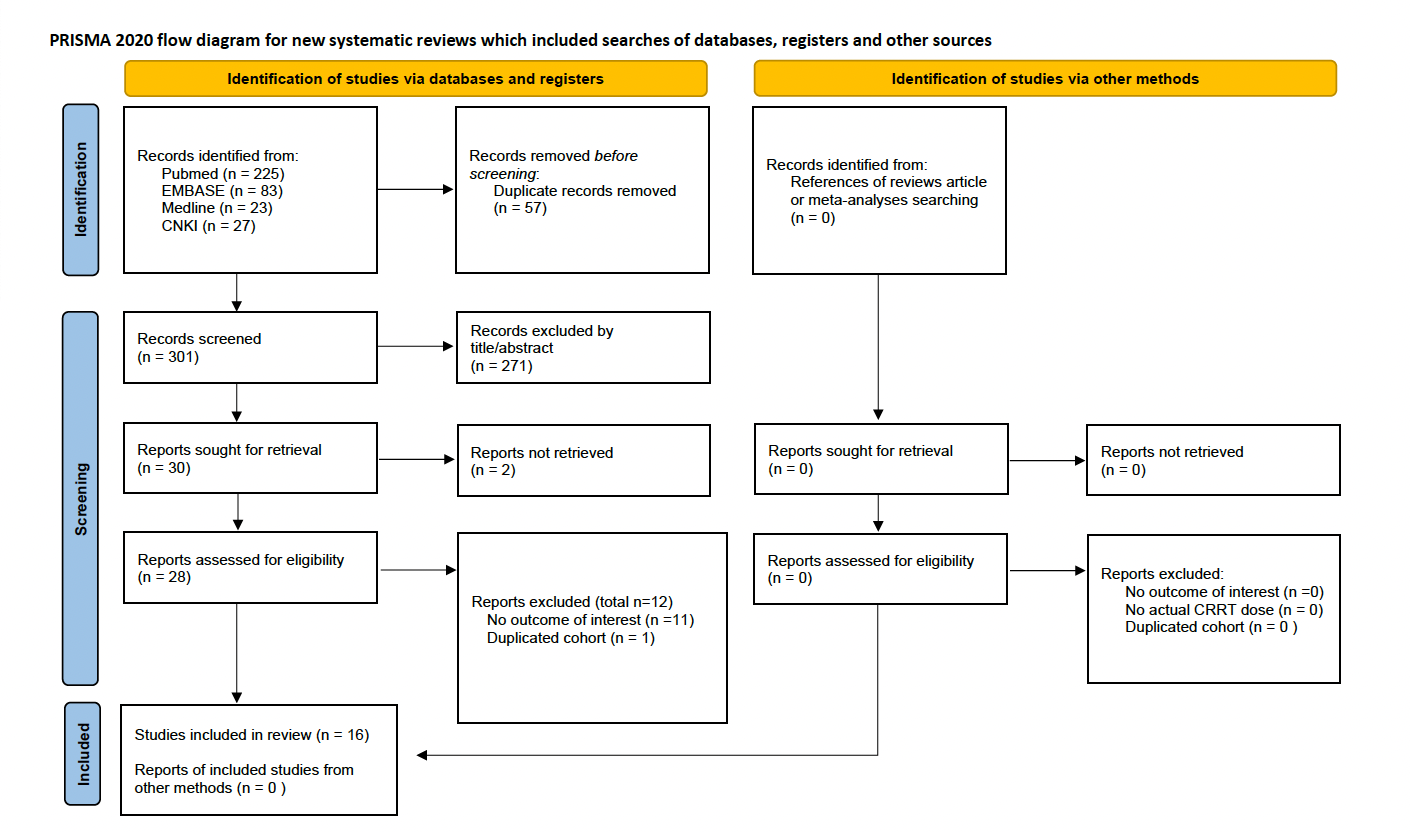
**

**Figure S2.** Fagan's nomogram for prognostic nutritional index as acute kidney injury prediction marker in non-CKD population with pre-test probability 15% (A), 25% (B) and 40% (C)

**
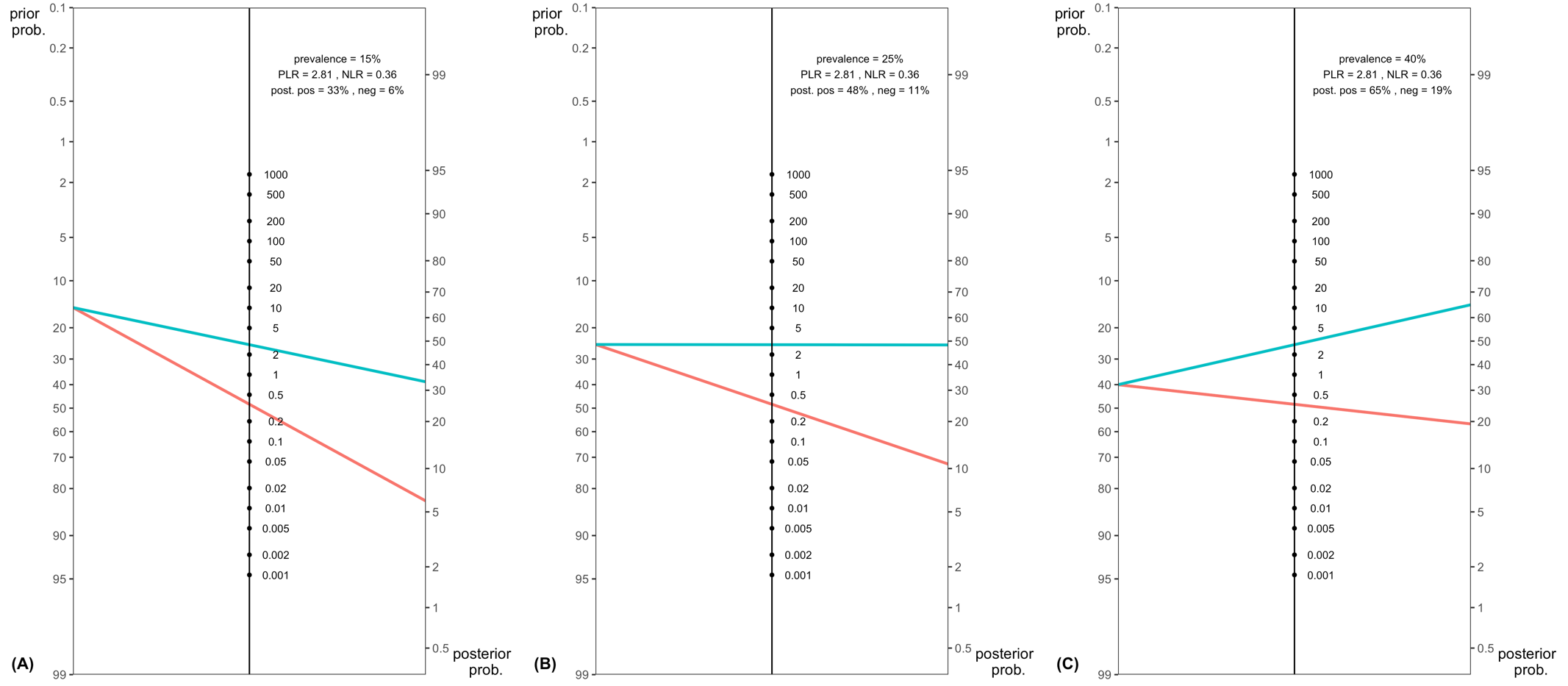
**

**Figure S3.** Deek’s funnel plot

**
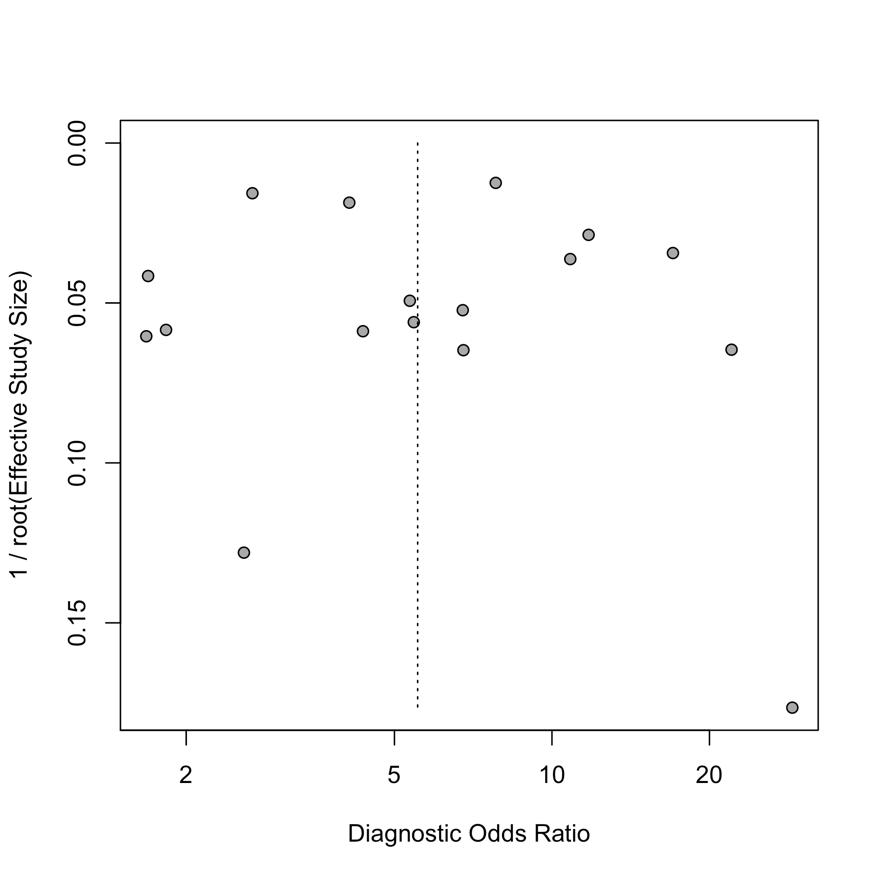
**

**Figure S4.** Assessment (A) and Summary (B) of risk of bias and applicability concern

**
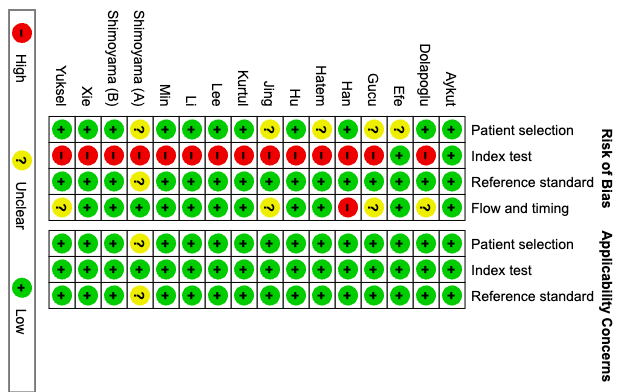

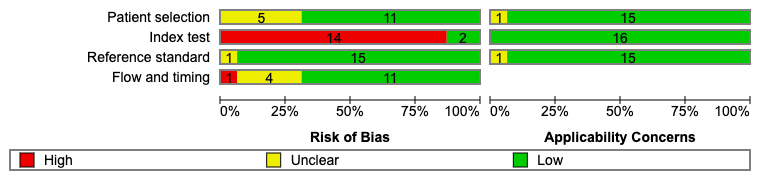
**
